# Chain-aware graph neural networks for molecular property prediction

**DOI:** 10.1093/bioinformatics/btae574

**Published:** 2024-10-11

**Authors:** Honghao Wang, Acong Zhang, Yuan Zhong, Junlei Tang, Kai Zhang, Ping Li

**Affiliations:** School of Computer Science and Software Engineering, Southwest Petroleum University, Chengdu 610500, China; School of Computer Science and Software Engineering, Southwest Petroleum University, Chengdu 610500, China; School of Computer Science and Software Engineering, Southwest Petroleum University, Chengdu 610500, China; College of Chemistry and Chemical Engineering, Southwest Petroleum University, Chengdu 610500, China; School of Computer Science and Technology, East China Normal University, Shanghai 200062, China; School of Computer Science and Software Engineering, Southwest Petroleum University, Chengdu 610500, China

## Abstract

**Motivation:**

Predicting the properties of molecules is a fundamental problem in drug design and discovery, while how to learn effective feature representations lies at the core of modern deep-learning-based prediction methods. Recent progress shows expressive power of graph neural networks (GNNs) in capturing structural information for molecular graphs. However, we find that most molecular graphs exhibit low clustering along with dominating chains. Such topological characteristics can induce feature squashing during message passing and thus impair the expressivity of conventional GNNs.

**Results:**

Aiming at improving node features’ expressiveness, we develop a novel chain-aware graph neural network model, wherein the chain structures are captured by learning the representation of the center node along the shortest paths starting from it, and the redundancy between layers are mitigated via initial residual difference connection (IRDC). Then the molecular graph is represented by attentive pooling of all node representations. Compared to standard graph convolution, our chain-aware learning scheme offers a more straightforward feature interaction between distant nodes, thus it is able to capture the information about long-range dependency. We provide extensive empirical analysis on real-world datasets to show the outperformance of the proposed method.

**Availability and implementation:**

The MolPath code is publicly available at https://github.com/Assassinswhh/Molpath.

## 1 Introduction

Precise property predictions play a crucial role in the selection of chemical compounds with the desired attributes for subsequent tasks (David *et al.* 2020) within the realm of drug design and discovery, establishing itself as a pivotal task in this field. With the advent of deep learning techniques, molecular property prediction has achieved remarkable success (Wang *et al.* 2023b, Zhou *et al.* 2023). By encoding molecules as strings with some notation tools like SMILES (Weininger 1988) and SELFIES (Krenn *et al.* 2022), sequential models (Edwards *et al.* 2022, Irwin *et al.* 2022) can be employed to further extract higher-order features from the string for the downstream property classification and/or regression.

However, the one-dimensional string representation does not contain the information about atom-to-atom interactions, resulting in sub-optimal performances. To leverage the irregular structural feature of atom-to-atom interaction, recent work (Li *et al.* 2022a, Stärk *et al.* 2022, Zhu *et al.* 2022) resorts to graph neural networks, which models molecules as graphs by representing atoms as nodes and chemical bonds as edges. This way, the nonlinear interactions between atoms are able to be captured. In particular, it has been noticed by more recent work (Zang *et al.* 2023) that there are some unique structural patterns in molecules, e.g. rings and functional groups, suggesting that specific graph neural network models are required to obtain better feature representation. HiMol (Zang *et al.* 2023) shows that hierarchical architecture is effective for graph neural networks to capture different level of chemical semantic information.

**Figure 1.**
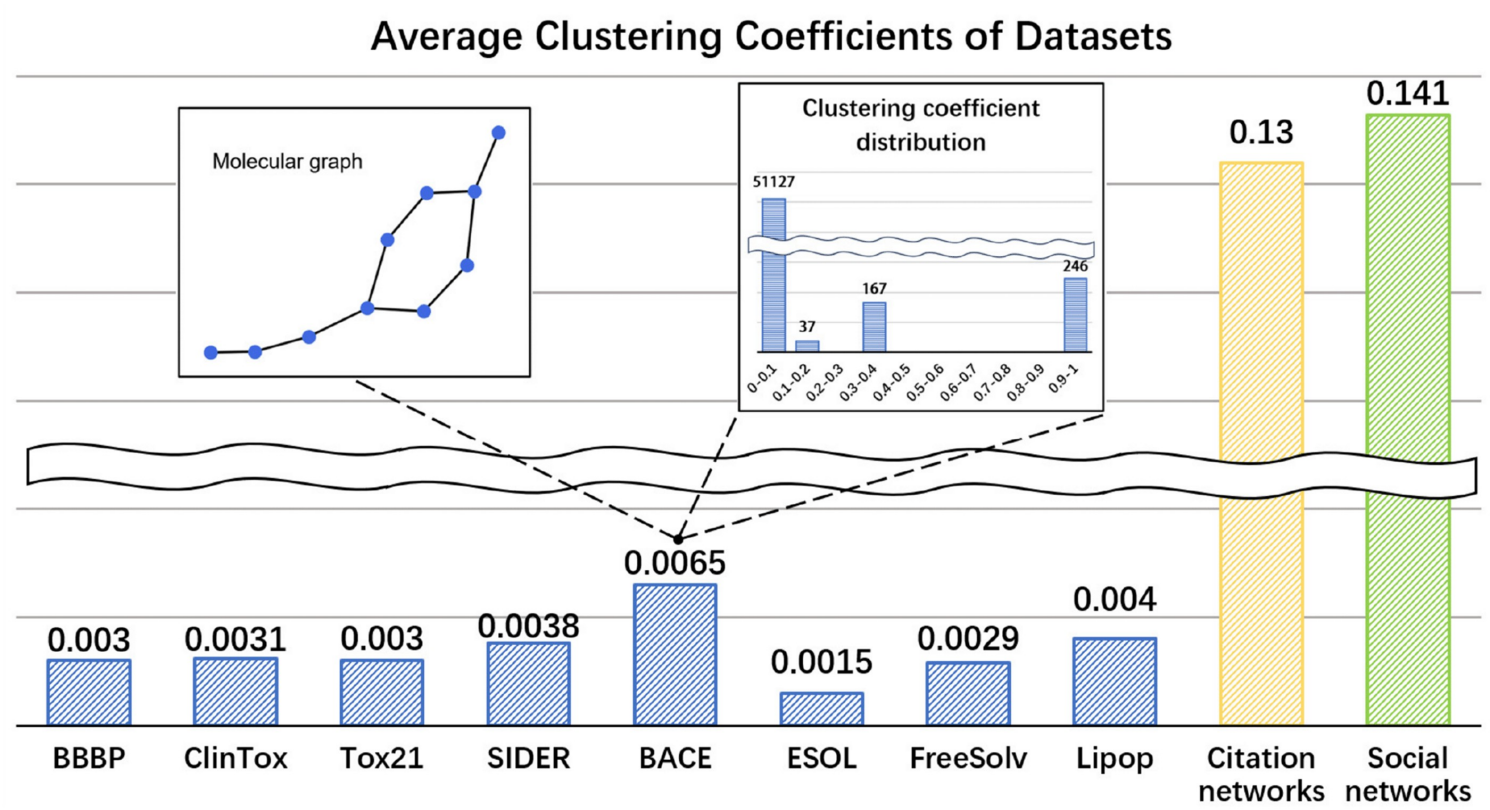
Average clustering coefficient of datasets. A sample molecule from dataset BACE is shown in the left side inset and the clustering coefficient distribution of dataset BACE is shown in the right-side inset.

Despite the local patterns (i.e. some motifs) implied in molecular graphs at mesoscopic level, the global properties also play a critical role in graph-based representation learning. Since most of current GNNs intrinsically perform local smoothing (Huang *et al.* 2023a, 2023b, 2024), limiting graph convolutions in the local regions around center nodes, it is hard for this scheme to capture the long-range dependency between nodes. When applying GNNs on molecular graphs, this drawback becomes more prominent, as the backbone of molecular graphs are generally chain-like. To get deep insight into the structural features of molecular graphs, we empirically study the local connectivity with clustering coefficient (Hamilton *et al.* 2017) that counts how likely the neighbors of a node are connected. Figure 1 demonstrates that there are only a very small number of nodes whose neighbors are well-connected, while for most nodes there are a lack of shortcuts between them, as shown in the inset of Fig. 1. This characteristic implies that information can only be transmitted along long-distance path between the majority of the nodes, which is considered as the root cause of over-squashing (Giraldo *et al.* 2023), a phenomenon that featural information on distant neighbors will be squashed during message passing. Clearly, the particular structure properties of molecular graphs call for rethinking the architecture of graph neural network models.

Ideally, an appropriate graph neural network is able to exploit the influences of long-range neighbors to enrich feature representation and avoid the side effect of message passing. Toward this end, we introduce a path-based graph learning framework, which we term as MolPath. Specifically, our model account for the effects of chain-like structure by integrating shortest paths between center node and its high-order neighbors at different levels (i.e. lengths of the shortest paths). To distinguish between shortest paths of different orders, we adopt attention mechanism to learn the path importance. Besides, considering there may exist information redundancy between different orders, e.g. the shortest path of length *k* may include the shortest paths of length i<k, we leverage the initial residual difference connection trick to update the representation of higher-order shortest paths. The representation of entire graph is then obtained by pooling all weighted shortest paths of different orders.

In summary, the contributions of this article are as follows:

- We develop a novel chain-aware graph neural network model, which imposes the message passing along the shortest paths between nodes, endowing the model with the ability to capture the chain-like backbone structure of molecular graphs.
- By employing initial residual difference connection (IRDC) trick on the shortest path convolution, MolPath alleviates the information redundancy between layers, thus optimizing feature representation of the nodes. In aggregating all paths with different lengths initiated at center node, MolPath opts for attention mechanism for weighted node fusion.
- We evaluate the proposed method on molecular property prediction (i.e. classification and regression) on several benchmark biological datasets. The experimental results demonstrate the superiority of our method over currently strong baseline models on both tasks.

The rest of this paper is organized as follows. Section 2 briefly introduces some related work. Section 3 details the experimental dataset. Section 4 presents our new model. Section 5 presents experimental results. Finally, Section 6 draws out conclusions.

## 2 Related work

There are some ways to learn representations for molecules, which can be roughly categorized into two types: GNN-based and SMILES string-based machine learning models.

### 2.1 SMILES string-based model

The dawn of the Transformer (Vaswani *et al.* 2017, Devlin *et al.* 2019) sparked a surge of interest to explore its application in encoding molecular data, such as GROVER (Rong *et al.* 2020) and ChemCrow (Bran *et al.* 2023). The input of these models are SMILES strings. Besides, CLAPS (Wang *et al.* 2023a) performs data enhancement on encoded SMILES strings through different mask methods combined with the attention mechanism. However, SMILES strings are one-dimensional data and therefore cannot capture complex structural information of molecules, which may pose limitations for certain tasks.

### 2.2 GNN-based model

This type of methods translate SMILES string into a graph and leverage the power of GNNs to learn the global representation of the graph, e.g. GraphMVP (Liu *et al.* 2021), MolCLR (Wang *et al.* 2022), and 3Dinfomax (Stärk *et al.* 2022), respectively, use different GNN methods to encode molecular data and then fuse the encoded features under a contrastive learning framework. HiMol (Zang *et al.* 2023) adopts a unique molecular decomposition strategy to decompose the molecular graph into motifs according to chemical rules such as the BRICS (Degen *et al.* 2008) algorithm to better capture the topology and unique structure of the molecular graph. This method is unique in that it can analyze the structure of a molecule at multiple levels. However, there are some drawbacks with the above models. For instance, contrastive learning-based models usually perform data augmentation by randomly deleting and adding nodes or edges, which may change the chemical structure of the molecule, thereby destroying the chemical semantics within the molecule. On the other hand, for the data whose clustering coefficient is relatively low, simply capturing unique structures, such as rings, may not reflect the complete structural information of the molecular graph well. To resolve this issue, we propose a chain-aware GNN model for long-distance dependency learning.

## 3 Materials

To evaluate the effectiveness of the proposed MolPath model compared to existing molecular representation learning methods, we predict properties of molecules on eight benchmark datasets sourced from MoleculeNet (Wu *et al.* 2018) across multiple domains, namely, physicochemical datasets (i.e. ESOL, FreeSolv, and Lipo) for regression tasks, physiological datasets (i.e. BBBP, Tox21, SIDER, ClinTox), and biophysical dataset BACE for classification tasks.

For regression task, the output of MolPath corresponds to the molecular property value. For classification, the model’s output consists of categorical property labels. Furthermore, each dataset is partitioned into training, validation, and test subsets at an 8:1:1 ratio. The training subset is dedicated to training the model, while the validation subset serves the purpose of fine-tuning hyperparameters. The test subset is employed to evaluate the model’s overall performance.

A comprehensive overview of the primary dataset statistics is presented in Table 1.

**Table 1.** Summary information of the MoleculeNet benchmark datasets.

Category	Dataset	Tasks type	# Tasks	# Compounds	Metric
Physical chemistry	ESOL	Regression	1	1128	RSME
	FreeSolv	Regression	1	642	RSME
	Lipop	Regression	1	4200	RSME
Physiology	BBBP	Classification	1	2039	ROC-AUC
	Tox21	Classification	12	7831	ROC-AUC
	SIDER	Classification	27	1427	ROC-AUC
	ClinTox	Classification	2	1478	ROC-AUC
Biophysics	BACE	Classification	1	1513	ROC-AUC

## 4 Methods

### 4.1 Framework

**Figure 2.**
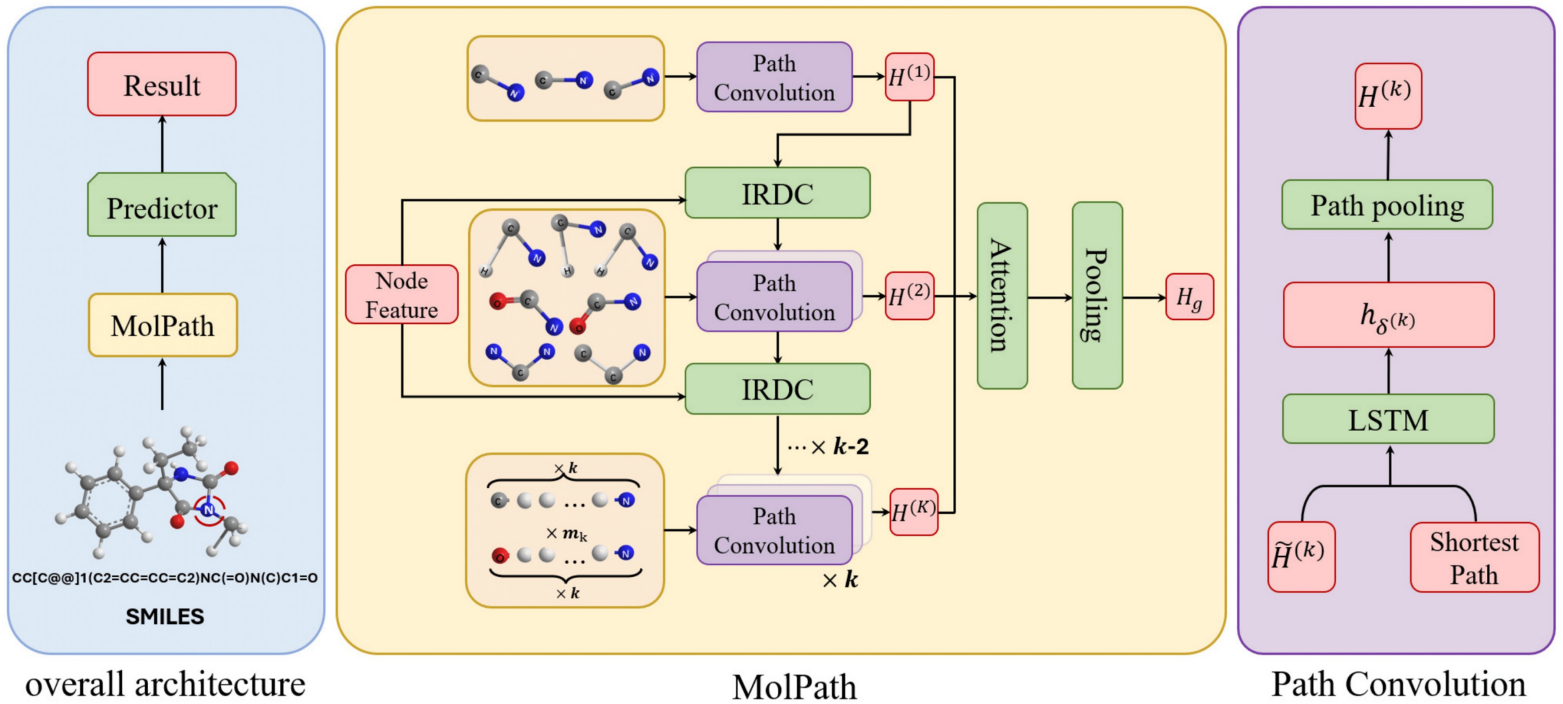
The framework of our method MolPath. Left: The overall architecture. Middle: Details in the MolPath. Right: Details in the Path Convolution block.

The architectural framework of our approach, referred to as MolPath, is visually represented in Fig. 2. The core of our proposed model is to learn the feature representation of the entire molecular graph by the designed MolPath module, which first embeds node features and then aggregate them with attention scores. Finally, the node features are compressed to be row vector for the downstream prediction.

### 4.2 Notation

Let G=(V,E) be an undirected graph consisting of a set of nodes *V* and a set of edges E⊆V×V, where |V|=n nodes and |E|=m edges. The set $N_{\left(v\right)}$ represents the neighbors of node *v*. For attributed graphs, each node v∈V is endowed with an initial node feature vector denoted by $X_{v}\in R^{d}$, which can contain categorical or real-valued properties of node *v*. Moreover, we denote the set of paths of length *k* by $\delta^{\left(k\right)}=\left\{\delta_{1}^{\left(k\right)},\delta_{2}^{\left(k\right)},\ldots ,\delta_{m_{k}}^{\left(k\right)}\right\}$, where $m_{k}$ is the number of shortest paths with length *k*. Note that paths only contain distinct nodes. A path is a sequence of nodes $\delta_{i}^{\left(k\right)}=\left[v_{1},v_{2},\ldots ,v_{k+1}\right]$, in which there is an edge linking one node in the sequence to its successor in the sequence, with no repeated nodes. Formally, the path starting at node *i* is denoted as $v_{i}$, where for any two consecutive nodes *j* and j+1, $E_{j,j+1}=1$ and j≠s for any two different nodes *j* and *s*. When k=0, there is only one source node in the path, $\delta^{\left(0\right)}$ represents the nodes, and $H^{\left(0\right)}\in R^{n\times d}$ is the initial feature vector of nodes.

### 4.3 Path convolution

In the following, we demonstrate each component in details. We first calculate the shortest path $\delta^{\left(k\right)}$ of a given length *k*. Then our path convolution will perform along the shortest paths originated at the central node, which is different from the vanilla graph convolution (Kipf and Welling 2016) that involves recursive message passing. In particular, for each node presentation at the *k*th convolutional layer, to reduce the redundancy between layers, we introduce initial residual connections (IRDC) module to learn the representation difference for each node between its shortest path with different lengths.

#### 4.3.1 Initial residual connections

**Figure 3.**
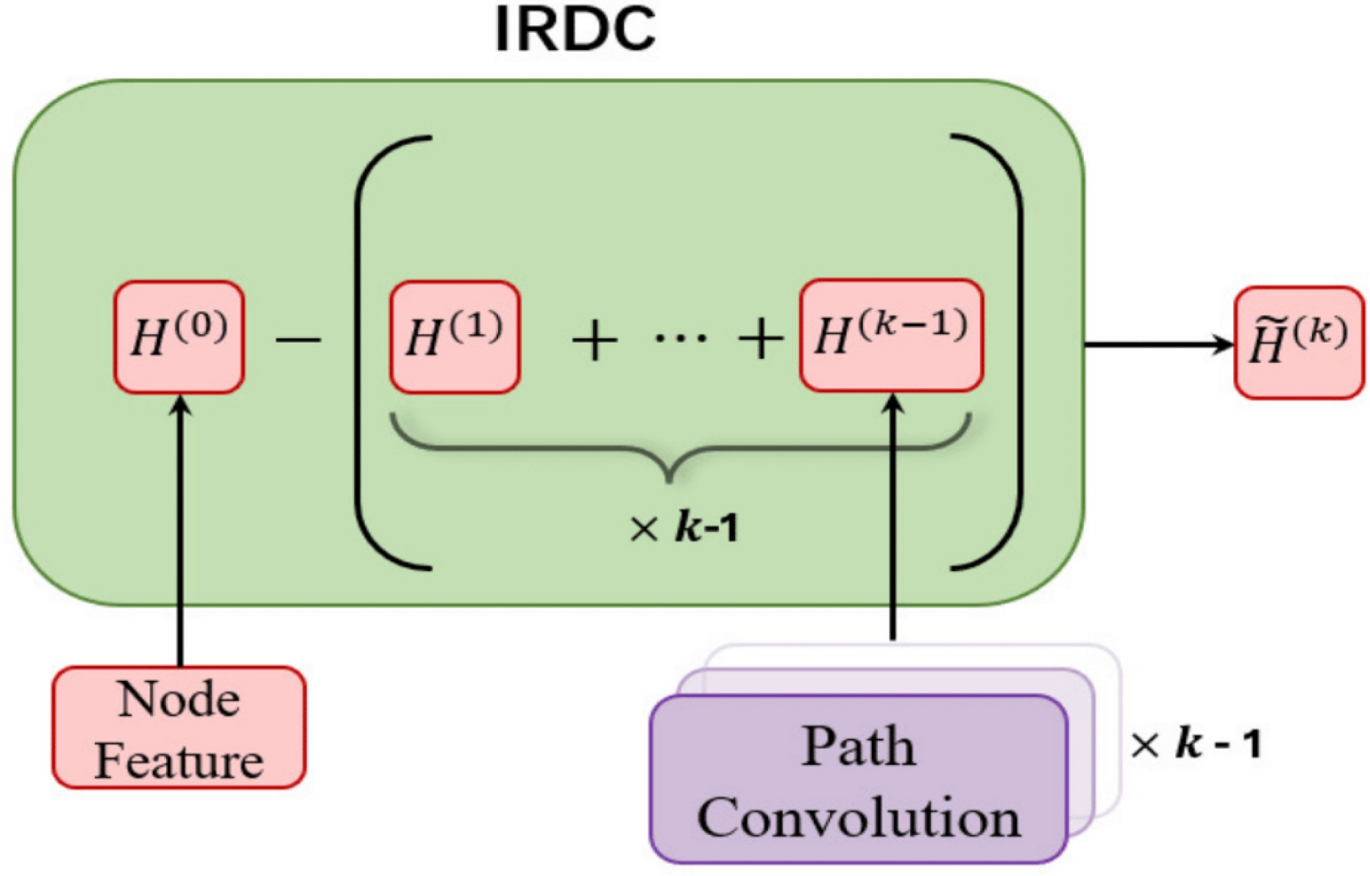
An illustrative example of Initial Residual Difference Connection (IRDC) when path length is k−1. The ${\tilde{H}}^{\left(k\right)}$ is the input for the convolution with path length *k*.

As shown in Fig. 3, IRDC accounts for the difference between the initial node feature and the representations learned at previous convolution layers, as the information gain at the current layer. This operation attempts to capture as much as possible novel features at each layer. Since our convolution is performed along the shortest paths, while the shortest paths of length k−1 are included in the shortest paths of length *k*, this implies that convolution on paths of length *k* inevitably contains information about paths of length k−1. It necessitates the feature redundancy reduction using IRDC. Therefore, at layer *k*, before applying LSTM on the node sequences, we calculate the IRDCs for the nodes:

(1) $$\begin{array}{c} \begin{array}{c} \begin{array}{cc} {\tilde{H}}_{\delta_{i}^{\left(k\right)}} & =\mathrm{IRD}C^{\left(k\right)}(H_{\delta_{i}^{\left(k\right)}}^{\left(0\right)},\;\sum_{j=1}^{k-1}H_{\delta_{i}^{\left(k\right)}}^{\left(j\right)})\\ & =(1-\lambda )H_{\delta_{i}^{\left(k\right)}}^{\left(0\right)}-\lambda \sum_{j=1}^{k-1}H_{\delta_{i}^{\left(k\right)}}^{\left(j\right)}, \end{array} \end{array} \end{array}$$

where *k* denotes the length of the shortest path, and λ∈(0,1] is a hyperparameter. The term $\sum_{j=1}^{k-1}H_{\delta_{i}^{\left(k\right)}}^{\left(j\right)}$ in Equation (1) can be seen as the total information extracted by the previous k−1 layers. This mechanism enables model to fully exploit the distinguished features embodied in the original attributes and the features acquired through convolutions. To address potential issues related to value scaling, we perform batch normalization on node representations.

Next, we further learn the sequential correlation among nodes in a path, by employing Long Short-Term Memory(LSTM) (Hochreiter and Schmidhuber 1997)

(2) $$H_{\delta_{i}^{\left(k\right)}}=\mathrm{LSTM}({\tilde{H}}_{\delta_{i}^{\left(k\right)}}^{\left(k\right)},\;\delta_{i}^{\left(k\right)}),$$

where $H_{\delta_{i}^{\left(k\right)}}$ are the updated representations of the nodes on the *i*-th path of length *k* $\delta_{i}^{\left(k\right)}$ after performing sequence learning.

We note that, the LSTM operates on reversed paths, as we hypothesize that the most important information in the sequence is contained in the representation of starting node.

Then the final node representation at layer *k* is the aggregation of all the shortest paths of length *k* starting at the central nodes. In particular, we employ path pooling on individual paths. After normalization and a 2-layer MLP, we acquire the embeddings of the nodes at convolution layer *k*, i.e. $H^{\left(k\right)}\in R^{n\times d}$.

(3) $$H^{\left(k\right)}=\phi (\mathrm{NORM}(H^{\left(k-1\right)}+\sum_{i=1}^{m_{k}}H_{\delta_{i}^{\left(k\right)}})).$$

Accordingly, performing *L* layers of path convolution can obtain the information about the *L*th-order neighbors (i.e. *L* hops distancing from the central nodes).

### 4.4 Path attention and graph pooling

On the conjecture that different shortest paths have different influence on the node representation learning, we utilize attention mechanism on the shortest paths with different lengths to weigh each type of shortest path. We use the initial feature as the query for attention score computation:

(4) $$\omega_{i}=\mathrm{softmax}(\frac{{\left(W_{\mathrm{Att}}\cdot H^{\left(0\right)}\right)}^{T}\cdot (W_{\mathrm{Att}}\cdot H^{\left(i\right)})}{\sqrt{d}}),i=1,2,\ldots ,k,$$

Accordingly, by aggregating the initial feature and the weighted sum of all the shortest paths of different length originated from the central node, we obtain the node representations: $H^{\left(0\right)}+\sum_{i=1}^{k}\omega_{i}H^{\left(i\right)}$. The graph representation is then achieved by pooling all nodes

(5) $$H_{g}=\mathrm{Pool}(H^{\left(0\right)}+\sum_{i=1}^{k}\omega_{i}H^{\left(i\right)}),$$

For downstream property prediction task, we use a two-layer MLP to generate the prediction results:

(6) $$\hat{y}=\phi (H_{g}).$$

### 4.5 Property prediction loss

We choose different loss functions according to different molcular prediction tasks. Specifically, for classification task, the binary cross entropy is used:

(7) $$\begin{array}{c} \begin{array}{cc} \mathrm{BCE}(y,\hat{y}) & =-1/n\sum_{i}(y_{i}\times \text{log}({\hat{y}}_{i})\\ & +(1-y_{i})\times \text{log}(1-{\hat{y}}_{i})), \end{array} \end{array}$$

where *y* is the target label, $\hat{y}$ is the predicted label that is first processed by the sigmoid function, *n* indicates the number of total labels.

As for regression task, we utilize the L1−loss:

(8) $$L(y,\hat{y})=1/n\sum_{i=1}^{n}\left|y_{i}-\hat{y_{i}}\right|,$$

where *y* is the real value, $\hat{y}$ is the predicted value.

### 4.6 Performance metrics

As suggested by MoleculeNet (Wu *et al.* 2018), ROC-AUC is used as the performance metric for classification on datasets BBBP, Tox21, SIDER, ClinTox, and BACE. On the three regression datasets ESOL, FreeSolv, and Lipop, we use RMSE to evaluate the performance.

### 4.7 Time complexity

To enumerate the shortest paths from the source node to all other nodes of length at most *K*, we use the Depth First Search (DFS) algorithm. This takes at most $O(b^{K})$, where b is the nodes’ degree and the upper bound of *b* is the maximum nodes’ degree. For all nodes in the graph, the time complexity is $O(nb^{k})$.

Molecules graphs are often small and/or sparse, *n* is often small and/or b≪n. Thus, for bounded *K*, the time complexity for enumerating the paths is not prohibitive.

Our model obtains path representation through LSTM, whose time cost is $O(td^{2})$, where *t* is the length of path of the current layer and *d* is the hidden layer dimension. Moreover, the time complexity for the path attention mechanism is denoted as $O(nd^{2})$, where *n* is the number of nodes. Consequently, the time complexity of the model is $O(nb^{k}+Ktd^{2}+nd^{2})$, where *K* is the number of iterations of path convolution.

## 5 Results

### 5.1 Baselines and experimental settings

We comprehensively evaluate our method and compare it against ten methods:

- GCN (Kipf and Welling 2016), GIN (Xu *et al.* 2018), which are commonly used GNNs for graph representation learning.
- GROVER (Rong *et al.* 2020): It can learn rich structural and semantic information of molecules from enormous unlabeled molecules. Rather, to encode such complex information, GROVER integrates message passing networks into the transformer-style architecture to deliver a class of more expressive encoders of molecules.
- GraphMvp (Liu *et al.* 2021): A molecular property prediction model employs a contrastive learning framework that exploits consistency between 2D topological structures and 3D geometric views to enhance molecular representations, ultimately improving the predictive performance of downstream task models in MoleculeNet.
- GEM (Fang *et al.* 2022): A new geometry-enhanced molecular representation learning method is proposed to capture the geometric information and topological information of molecules.
- GeomGcl (Li *et al.* 2022b): Propose a novel graph contrastive learning method utilizing the geometry of the molecule across 2D and 3D views.
- HiMol (Zang *et al.* 2023): By encoding motif structure and extracting motif hierarchical graph representations, coupled with multi-level self-supervised pre-training (MSP), learn molecular representations and predict molecular properties.
- 3D PGT (Wang *et al.* 2023b): A new 3D pre-training framework pre-trains the model on a multi-task pre-training framework containing three attributes: Bond length, bond angle, and dihedral angle, and then trains the model on a molecular graph without a 3D structure. Fine-tune it.
- Uni-mol (Zhou *et al.* 2023): A universal 3D molecular representation learning pre-training framework based on Graphormer, which can apply pre-trained models to various downstream tasks through several different fine-tuning strategies.
- 3DGCL (Moon *et al.* 2023): A small-scale 3D molecular contrastive learning framework that selects different conformers of molecules as positive samples.

According to different datasets, the parameter settings are also different. In our experiments, each dataset is splitted into three parts: training set, validation set, and test set. The optimal hyperparameters are obtained on the validation set by performing grid search, a commonly used hyper-parameter tuning approach. To reduce the influence of randomness on the results, we run the methods four times on the test set of each dataset and report the averaged results. All experiments use the Adam optimizer and are performed on NVIDIA RTX3090 GPU. A summary of the main parameter settings in the training phase is shown in Table 2.

**Table 2.** Parameter settings in MolPath.

Parameter	Value
Batch size	16, 32, 64, 128
Dropout	0, 0.01, 0.02, 0.03
Learning rate	$10^{-5}$ , $10^{-4}$, $10^{-3}$
Hidden size	[128, 700]
λ	[0, 0.6]
K	[4, 12]

### 5.2 Performance evaluation and comparison

Table 3 presents the performance of MolPath on molecule property regression and classification datasets, respectively. For regression task, our method consistently outperforms all baseline methods across the three benchmark datasets and achieves a new state-of-the-art on average, demonstrating its effectiveness and superiority in predicting molecular properties. We note that, the improvement of our MolPath on the Lipop dataset is marginal. This is because Lipop has a higher clustering coefficient compared to other datasets, which introduces more structural complexity challenge to our chain-oriented method.

**Table 3.** Performance comparison on classification (measured by ROC-AUC and regression [measured by RMSE] tasks).^a^

Method	**ESOL** ↓	**FreeSolv** ↓	**Lipop** ↓	Average	**BACE** ↑	**BBBP** ↑	**ClinTox** ↑	**Tox21** ↑	**SIDER** ↑	Average
GCN	0.778	1.582	0.899	1.086	0.829	0.895	0.615	0.788	0.624	0.750
GIN	0.619	1.136	0.756	0.837	0.850	0.890	0.753	0.824	0.618	0.787
GROVER	0.983	2.176	0.817	1.325	0.826	0.700	0.812	0.743	0.648	0.746
GraphMvp	1.092	^b^	0.681	^b^	0.812	0.724	0.790	0.631	0.639	0.719
GEM	0.798	1.877	0.660	1.109	0.856	0.724	0.906	0.781	0.672	0.788
GeomGcl	0.764	0.877	0.544	0.728	^b^	^b^	0.917	0.851	0.647	^b^
HiMol	0.833	2.283	0.708	1.275	0.846	0.732	0.808	0.762	0.625	0.755
3D PGT	1.061	^b^	0.687	^b^	0.809	0.721	0.794	0.738	0.606	0.734
Uni-miol	0.788	1.48	0.603	0.957	0.857	0.729	**0.919**	0.796	0.659	0.792
3DGCL	0.778	1.441	^b^	^b^	0.792	0.855	^b^	^b^	^b^	^b^
**MolPath(ours)**	**0.266**	**0.200**	**0.524**	**0.330**	**0.898**	**0.913**	0.863	**0.868**	**0.674**	**0.843**

a The SOTA results are shown in bold. The underlined is the second best.

b The result is unavailable in the original paper.

For classification task, we can observe that MolPath surpasses all competing methods on four out of the five datasets. As a whole, MolPath is definitely superior to the baselines in terms of average performance. Another observation is that by leveraging 3D structure information, Uni-mol performs well on graph classification, especially on ClinTox and BACE that have higher clustering coefficients. These results suggest that our method is able to learn the chain-like backbone for molecule graphs, but limited on cycle-rich topology.

However, compared to HiMol, the model specialized in discerning specific structural components (i.e. motifs) within molecules, our method shows consistent superiority across different task. In particular on regression task, the chain-aware MolPath outperforms motif-oriented HiMol by a large margin. This phenomenon may suggest from another angle that a majority of molecule graphs are low-clustering, which coincides with our empirical findings, as shown in Fig. 1. Accordingly, it is practically useful to learn chain structures rather than cycle-involved motifs.

### 5.3 Visualization of molecular representations

**Figure 4.**
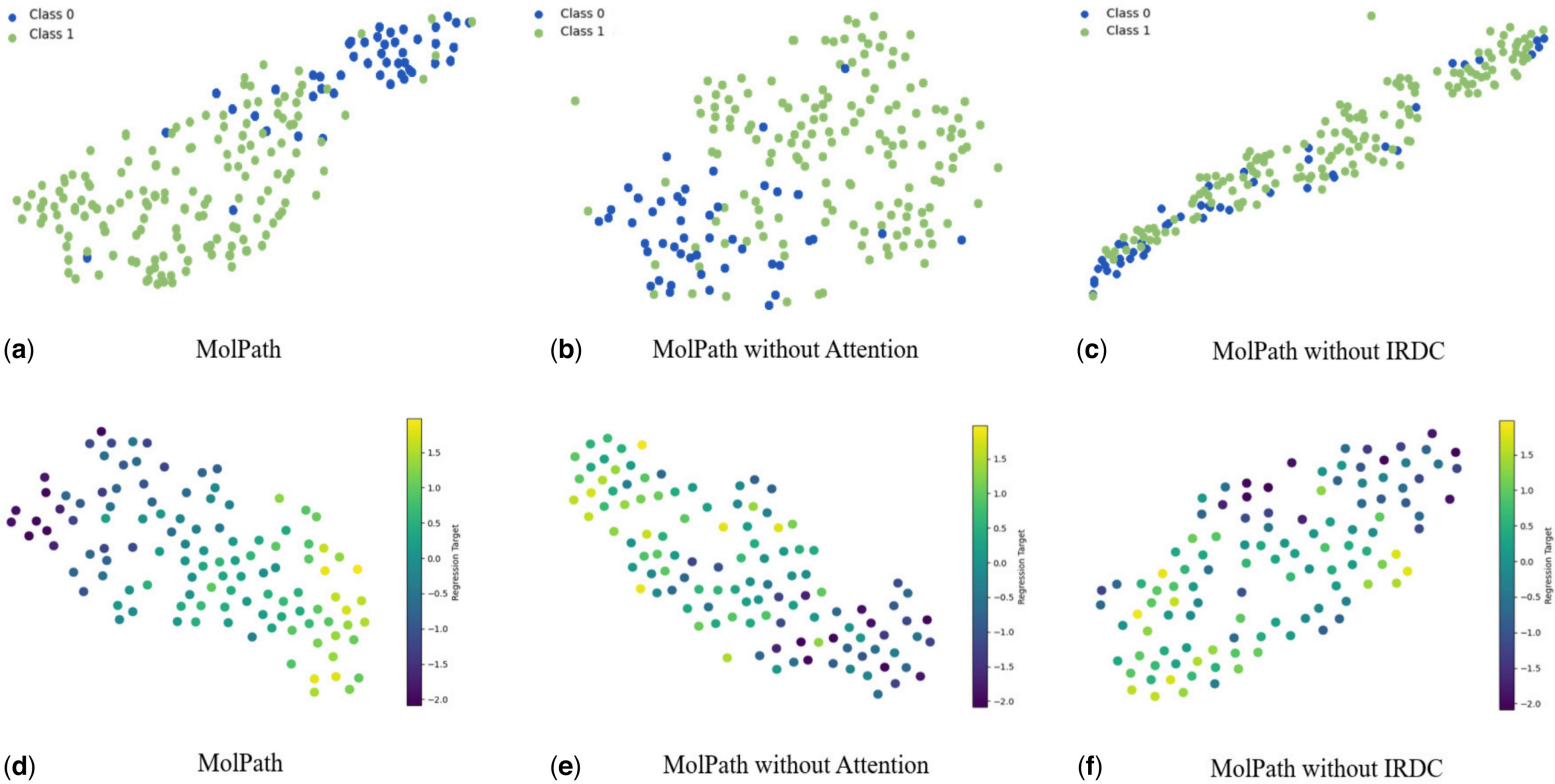
t-distributed Stochastic Neighbor Embedding (t-SNE) visualization of BBBP and ESOL. (a)–(c) demonstrate the excellent effect of MolPath on classification tasks and confirm the effectiveness of the component on classification tasks. (d)–(f) demonstrate the excellent effect of MolPath on regression tasks and confirm the effectiveness of the component on regression tasks.

In order to explore the effectiveness of our MolPath visually, we first embed the testset of BBBP and ESOL into a latent space, and then use the classic dimensionality reduction method t-SNE (t-distributed Stochastic Neighbor Embedding, Van der Maaten and Hinton 2008) for visualization. The results are reported in Fig. 4, where Fig. 4a–c correspond for MolPath, MolPath without IRDC, and MolPath without attention, respectively. A clear observation in Fig. 4a is that data points’ separation in MolPath is significantly improved when all components are retained, surpassing other tow plots. In contrast, in both Fig. 4b and Fig. 4c, the two types of data points become intermingled, highlighting the compelling evidence for the superior classification performance of MolPath. Also in Fig. 4d–f, without removing any components, the trend of Molpath is more obvious, and points with similar regression values are also more clustered.

### 5.4 Ablation study

In this section, we perform ablation experiments on the aforementioned classification and regression datasets to investigate how various components of the model and different values of λ impact experimental performance.

**Figure 5.**
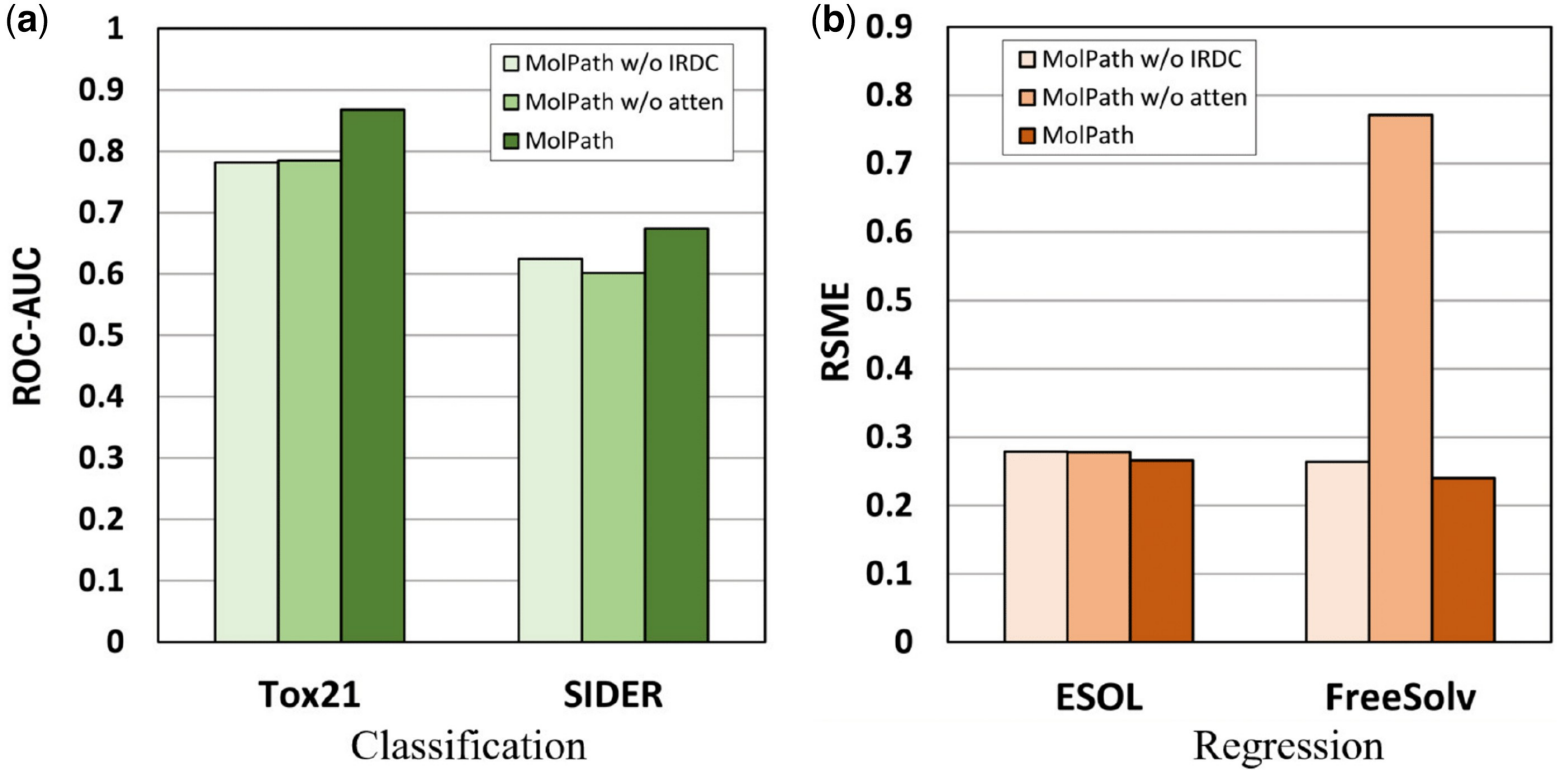
Ablation effect of MolPath on classification and regression datasets. (a) The performance of MolPath with different ablations on classification datasets. (b) The performance of MolPath with different ablations on regression.

Figure 5a and b shows the performance comparison of the models after sequentially removing various components on classification and regression datasets. Notably, when all components are retained, MolPath consistently achieves excellent results in both tasks. This emphasizes the model’s ability to obtain high-quality molecular representations and highlights its effectiveness in learning long-range node representations. In addition, the results demonstrate the ability of MolPath to capture and enhance chain graph representations through effective node pooling.

Next, we delve into the influence of the parameter λ in the context of Initial Residual Difference Connection (IRDC) and its impact on experimental performance. In our previous discussion, we introduced the significance of IRDC, which allows for the utilization of additional information that may not be initially harnessed within the node features during the convolution process. The magnitude of λ signifies the ratio between the initial node representation and the shortest path representation of different lengths.

**Figure 6.**
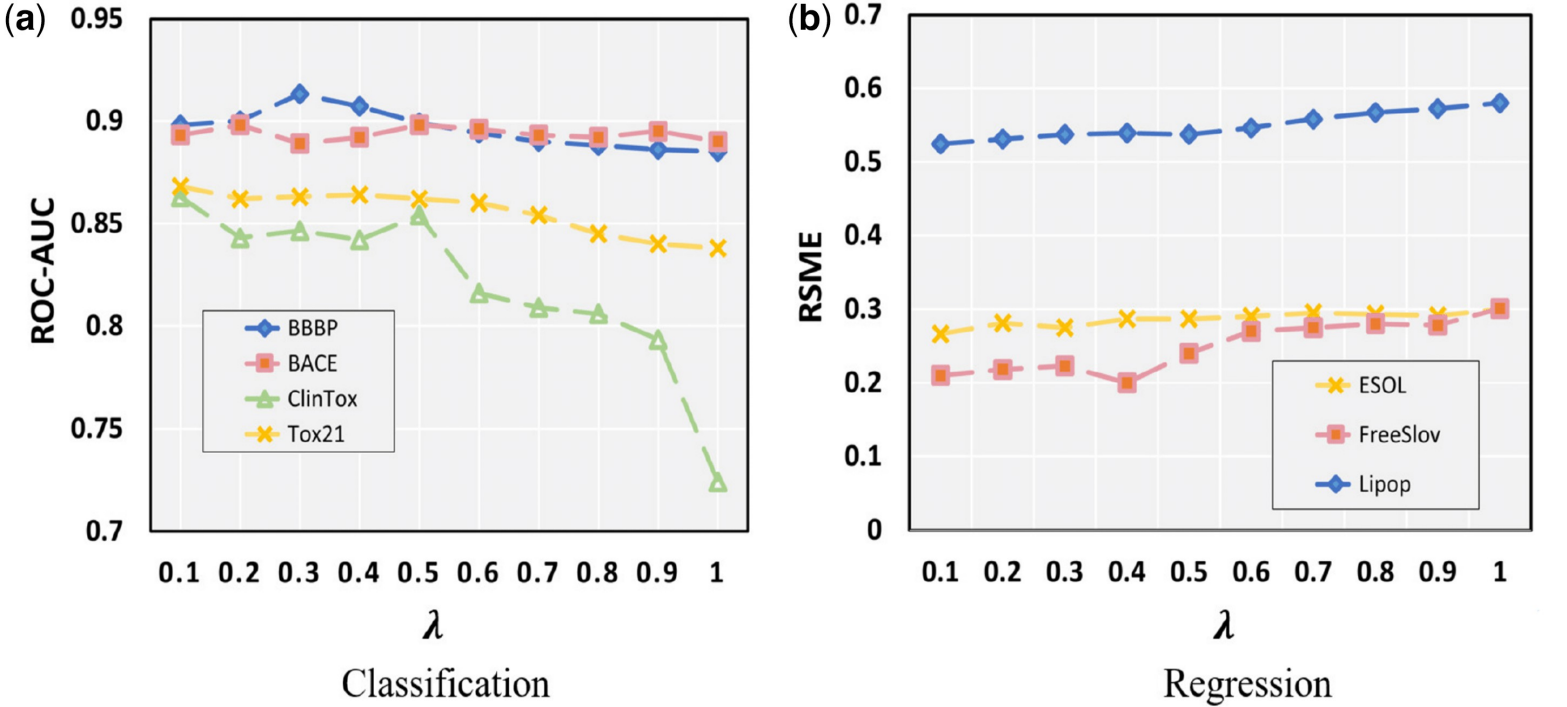
The performance of the model under different λ (a) Classification datasets; (b) Regression datasets.

As depicted in Fig. 6a, it is observed that an increase in the parameter λ leads to a decline in performance across the three classification datasets—BBBP, ClinTox, and Tox21. However, an intriguing deviation is noted in the BACE dataset, where performance improves with higher values of λ, consistent with the trends illustrated in Fig. 1. This improvement can be attributed to the BACE dataset’s intrinsic characteristics, such as a higher aggregation coefficient and a greater prevalence of ring structures in its initial representation. Similarly, Fig. 6b demonstrates that, in the context of regression tasks, performance also deteriorates with increasing λ. This observation suggests that the convolutional layers become more adept at capturing relevant information, thereby optimizing overall model performance as λ is modulated.

## 6 Conclusion

In this paper, we introduce a novel chain-aware graph neural network model called MolPath to capture the backbone of molecular graphs. In particular, MolPath uses IRDC module to reduce the information redundancy between the shortest paths with different lengths. To differentiate the impact of different nodes on the graph, we adopt attention mechanism for information synthesis. Extensive experiments show the consistent outperformance of MolPath over the state-of-the-art methods on two prediction tasks.

It is interesting to note that the the outperformance of MolPath on most molcule graphs and slightly inferior performance on individual dataset (i.e. ClinTox) compared to motif-based method HiMol can reflect the universality of low-clustering of molecule graphs. It is also revealed that combining two types of models might be more powerful for molecule graph representation learning. Besides, the performance of the model is relevant to the initial representation of the molecules. It is helpful to explore the influence of the spatial structure on the ultimate prediction accuracy in the future work.
